# Revealing the pH-dependent conformational changes in sol g 2.1 protein and potential ligands binding

**DOI:** 10.1038/s41598-024-72014-w

**Published:** 2024-09-11

**Authors:** Siriporn Nonkhwao, Doungkamol Leaokittikul, Rina Patramanon, Nisachon Jangpromma, Jureerut Daduang, Sakda Daduang

**Affiliations:** 1 Faculty of Pharmaceutical Sciences, Khon Kaen University, Khon Kaen, 40002 Thailand; 2 Department of Biochemistry, Faculty of Science, Khon Kaen University, Khon Kaen, 40002 Thailand; 3 Faculty of Associated Medical Sciences, Khon Kaen University, Khon Kaen, 40002 Thailand; 4 Protein and Proteomics Research Center for Commercial and Industrial Purposes (ProCCI), Khon Kaen University, Khon Kaen, 40002 Thailand

**Keywords:** Venom protein Sol g 2.1, Pheromone-binding proteins (PBPs), Protein–ligand binding, PH-induced ligand releasing, Molecular dynamics simulations, Biochemistry, Proteins

## Abstract

Sol g 2, a major protein found in the venom of the tropical fire ant (*Solenopsis geminata*), is well-known for its ability to bind various hydrophobic molecules. In this study, we investigate the binding activity of recombinant Sol g 2.1 protein (rSol g 2.1) with potential molecules, including (*E*)-β-Farnesene, α-Caryophyllene, and 1-Octen-3-ol at different pH levels (pH 7.4 and 5.5) using fluorescence competitive binding assays (FCBA). Our results revealed that Sol g 2.1 protein has higher affinity binding with these ligands at neutral pH. Relevance to molecular docking and molecular dynamics simulations were utilized to provide insights into the stability and conformational dynamics of Sol g 2.1 and its ligand complexes. After simulation, we found that Sol g 2.1 protein has higher affinity binding with these ligands as well as high structural stability at pH 7.4 than at an acidic pH level, indicating by RMSD, RMSF, Rg, SASA, and principal component analysis (PCA). Additionally, the Sol g 2.1 protein complexes at pH 7.4 showed significantly lower binding free energy (∆*G*_*bind*_) and higher total residue contributions, particularly from key non-polar amino acids such as Trp36, Met40, Cys62, and Ile104, compared to the lower pH environment. These explain why they exhibited higher binding affinity than the lower pH. Therefore, we suggested that Sol g 2.1 protein is a pH-responsive carrier protein. These findings also expand our understanding of protein–ligand interactions and offer potential avenues for the development of innovative drug delivery strategies targeting Sol g 2.1 protein.

## Introduction

The tropical fire ant (*Solenopsis geminata*) is one of the ubiquitous ant species in Thailand. Its venom also contains four major proteins, including Sol g 1, 2, 3, and 4^1^. Importantly, Sol g 2 is one of the major protein components in the venom^2^. Sol g 2.1 protein (GenBank: UYX46120.1) exhibits an 83.05% sequence identity with Sol i 2 (*S. invicta*)^3^. Both proteins comprise five α-helices and three intramolecular disulfide bridges, forming a hydrophobic cavity. Notably, Sol i 2 demonstrates strong binding affinity with hydrophobic molecules such as (*E*)-β-farnesene which is a well-known aphid alarm pheromone, and plant volatiles. Additionally, Sol i 2 protein can bind with α-Caryophyllene which is an anti-inflammatory and anti-cancer compound, and 1-octen-3-ol which is a human attractant biting insects such as mosquitoes^4^^‒^^6^. Moreover, Sol i 2 and Sol g 2.1 proteins have high affinity binding with analogs of ant trail pheromones like decane and undecane^3,4^.

**Fig. 1:**
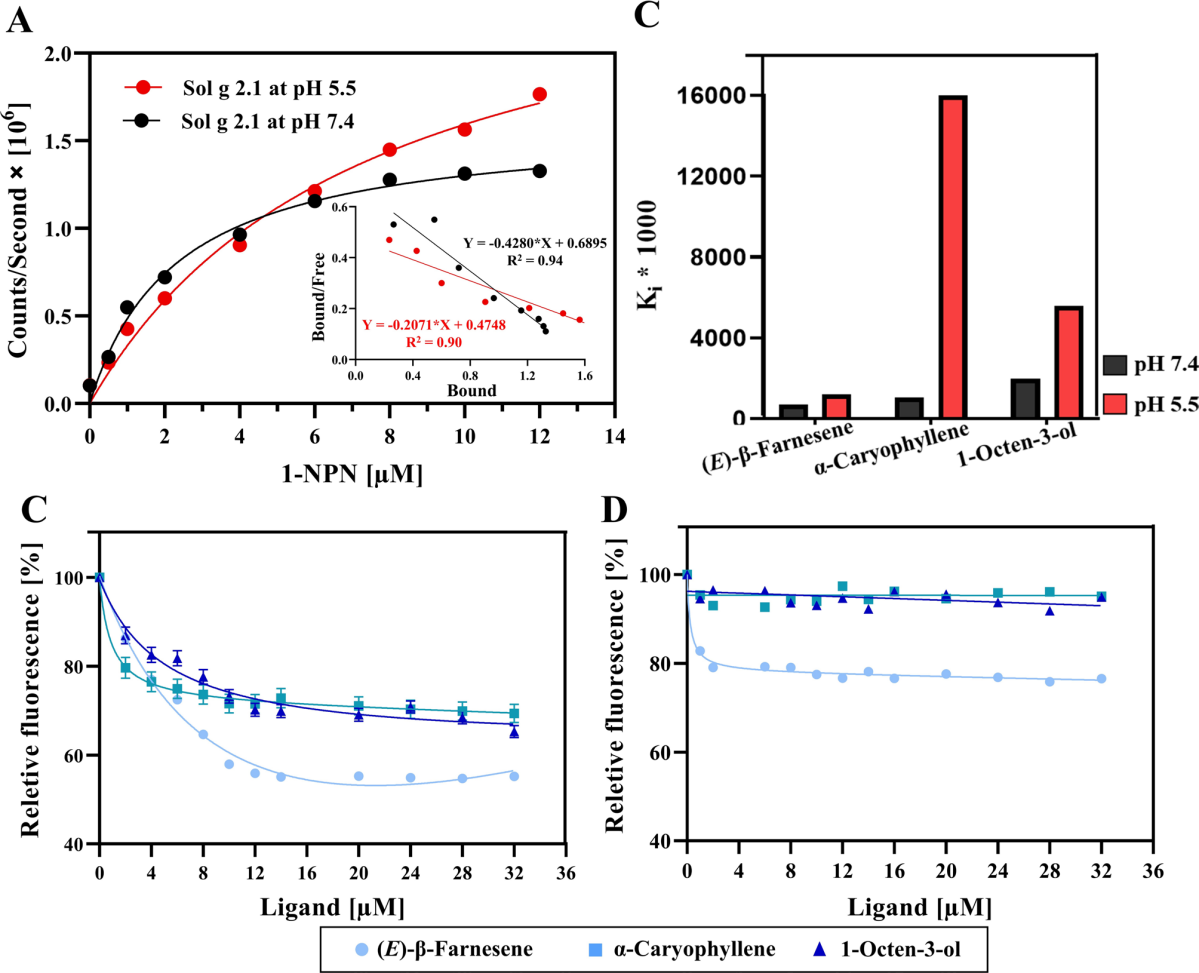
(**A**) The graph depicts the relationship between fluorescence intensity (Counts/second) and the concentration of 1-NPN aliquot added to rSol g 2.1 protein in Tris–HCl solutions at pH 7.4 (black line) and pH 5.5 (red line). Each point on the graph corresponds to the average fluorescence intensity at 400 nm maximal emission wavelength based on triplicates (± SEM). A nonlinear single-binding fitting model was utilized to fit the curve, and K_d_ values of 1-NPN and Sol g 2.1 at pH 7.4 and 5.5 were calculated from the Scatchard plots (insert). (**B**) Shows affinity constant (K_i_) of Sol g 2.1 and various ligands. Black and red columns represented the affinity constants at pH 7.4 and 5.5, respectively. (**C**‒**D**) Competitive binding curves of the ligands, including (*E*)-β-Farnesene, α-Caryophyllene and 1-Octen-3-ol ligands at pH 7.4 and 5.5, respectively. A combination of Sol g 2.1 and 1-NPN in 50 mM Tris–HCl buffer (at pH 5.5 and pH 7.4) underwent titration with 1 mM solutions of various competing ligands, reaching final concentrations ranging from 0 to 32 µM.

Interestingly, Sol g 2.1 closely resembles insect odorant-binding proteins (OBPs), which are small soluble proteins found in insect olfactory organs^7,8^. These proteins play a key role in the insect olfactory system by mediating between odorants and their membrane receptors^9^. This family includes pheromone binding proteins (PBPs), which specifically bind semiochemicals and transport pheromones to activate and protect pheromone receptors (PRs)^10^. Intriguingly, Sol g 2.1 and LmaPBP (PBP from the cockroach, *Leucophaea maderae*) share significant structural homology and possess an inner hydrophobic cavity^3^. Moreover, they share conserved binding residues which are crucial for the function of both proteins (as shown in Fig. 1S)^11^. Therefore, LmaPBP is a valuable template not only for understanding structural homology, but also for studying protein function. Hence, Sol g 2.1 protein may be involved in binding and transporting hydrophobic molecules, such as ant pheromones or straight-chain alkyl substituents of piperidine alkaloids as well as other potential molecules^4,12,13^. As mentioned, the structure of Sol g 2.1 protein is similar to OBPs which primarily serve the role of transporting chemical signals. A notable characteristic of most insect OBPs is their pH-dependent behavior, where they exhibit high affinity with their ligands at elevated pH levels but demonstrate reduced or no affinity at lower pH levels. The protein could potentially act as a transporter, releasing its ligands in response to the pH gradient at the cellular membrane such as insect sensilla lymph, neuronal, and cancer cell membrane, as indicated in prior studies^3,14–16^. Hence, we hypothesized that Sol g 2.1 protein may transport hydrophobic or potent molecules and subsequently bind to receptors on neuronal cell membranes or cell targets. This binding could result in the inhibition of neurotransmitter signaling through competitive binding and the induction of toxicity by these molecules^17–19^. Importantly, a previous report revealed that under acidic pH conditions, the aromatic amino acids of the Sol g 2.1 protein were exposed to the solvent, resulting in a transition of the protein from closed to open cavity binding^3^. Key amino acid residues that regulate pH-dependent ligand binding and release in the protein are crucial roles and lead to the loss of its rigid tertiary structure and a tendency to release its ligand-binding ability^20,21^. However, the specific key amino acids involved have not been identified yet.

Therefore, in this study, we first study the binding activity of recombinant Sol g 2.1 protein (rSol g 2.1) and various potential molecules, including (*E*)-β-Farnesene, α-Caryophyllene, and 1-Octen-3-ol at pH 5.5 and 7.4 by using fluorescence competitive binding assay (FCBA). We also predicted the structure of the protein and ligand complexes by molecular docking at different pHs. Moreover, insight into the stability determinants of proteins and ligand complexes was utilized by molecular dynamics simulations. To gain insight into the conformational changes of the Sol g 2.1 protein complexes were contributed by RMSD, RMSF, binding free energy, principal component analysis (PCA), and altering their protonation status. Furthermore, in this study, we purposed to discover a strategy for innovative methods in pest control and as a prospective approach for targeted drug delivery in the future.

## Results

### Competitive binding assay

**Fig. 2:**
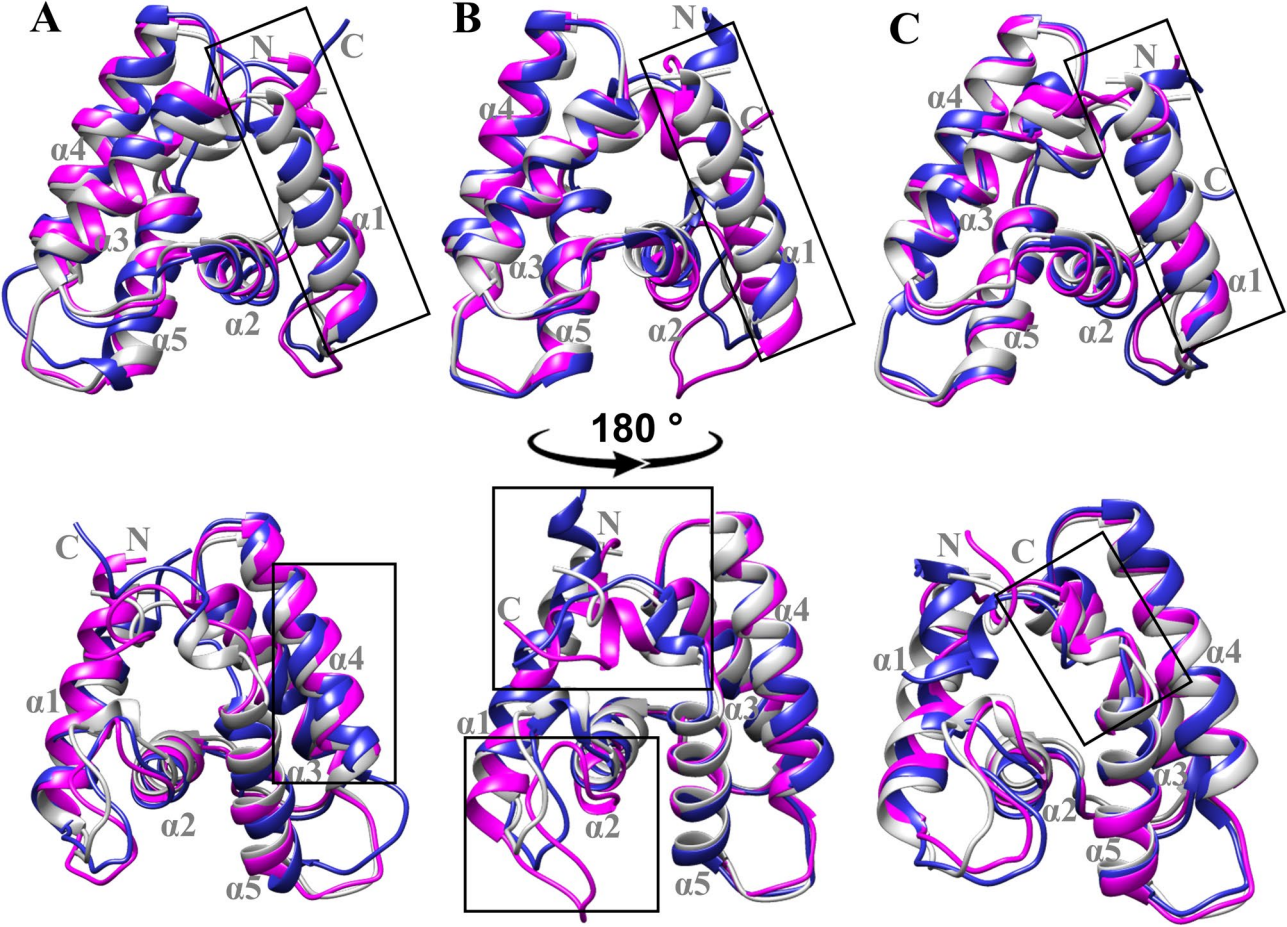
(**A**), (**B**), and (**C**) Show superimposed Sol g 2.1 protein structures after 100 ns simulation with (*E*)-β-Farnesene, α-Caryophyllene 1-Octen-3-ol, respectively at pH 7.4 (blue ribbon) and 5.5 (magenta ribbon) compared with the initial simulation of Sol g 2.1 protein (gray ribbon).

From the protein production, molecular weight of rSol g 2.1 protein was approximately 17 kDa. This discrepancy can be attributed to the presence of the 6X Histidine tag, which added approximately 1 kDa to the overall weight (as shown in Fig. 2S). To determine affinity binding, the K_d_ values of rSol g 2.1 protein at pH 5.5 and pH 7.4 with 1-NPN were calculated from the Scatchard plots. The results showed that K_d_ of rSol g 2.1 and 1-NPN at pH 7.4 and 5.5 were 2.33 ± 0.05 and 4.83 ± 0.03 µM (± SEM), respectively (as shown in Fig. 1A). This indicates that Sol g 2.1 protein has a high affinity for binding with 1-NPN at pH 7.4^22,23^. The reduction in fluorescence intensity at 400 nm was evaluated to assess the binding affinities of Sol g 2.1 protein with competitive ligands, including (*E*)-β-Farnesene, α-Caryophyllene, and 1-Octen-3-ol. The dissociation constant (K_i_) values of (*E*)-β-Farnesene, α-Caryophyllene, and 1-Octen-3-ol competitors with rSol g 2.1 protein at pH 7.4 were 0.72 µM (1.24 µM at pH 5.5), 1.06 µM (15.99 µM at pH 5.5), and 2.00 µM (5.61 µM at pH 5.5), respectively (as shown in Fig 1B; Table 1). The results of (*E*)-β-Farnesene, α-Caryophyllene, and 1-Octen-3-ol as the 1-NPN displacing ligands at different pH levels were shown as 1-NPN fluorescence intensity reduction (as shown in Fig. 1C‒D). These findings imply that the protein at the higher pH has the strongest affinity for binding with (*E*)-β-Farnesene, followed by α-Caryophyllene and 1-Octen-3-ol, respectively. In contrast, at pH 5.5, Sol g 2.1 protein showed the strongest binding with (*E*)-β-Farnesene, followed by 1-Octen-3-ol and α-Caryophyllene, respectively^24^. These results reveal that Sol g 2.1 protein has a suitable binding for the ligands at a higher pH than at a lower pH environment. Interestingly, this protein exhibits a significantly higher affinity binding with α-Caryophyllene at pH 7.4 than at pH 5.5, as indicated by a high K_i_ value. Thus, from these results, we believe that Sol g 2.1 protein has a specific binding with potential ligands at physiological pH and tends to release the ligand at lower pH, similar to the cancer cell membrane environment^25,26^.

**Table 1:** Binding affinities and top rank S score docking with the lowest RMSD (triplicate) of different ligands to Sol g 2.1 protein evaluated via competitive ligand binding assay and molecular docking (MOE software), respectively.

Ligands	CAS no	Purity (%)	pH 7.4			pH 5.5		
			S score^a^	RMSD (Å)^a^	K_i_ (µM)^b^	S score^a^	RMSD (Å)^a^	K_i_ (µM)^b^
(*E*)-β-Farnesene	18,794–84-8	96.6	− 7.64	0.84	0.72	− 7.01	1.29	1.24
α-Caryophyllene	6753–98-6	95.2	− 6.35	0.69	1.06	− 6.10	1.22	15.99
1-Octen-3-ol	3391–86-4	99.9	− 6.49	1.41	2.00	− 6.14	0.92	5.61

^a^Show the best S score from molecular docking using MOE version 2019 (Molecular Operating Environment) after run for triplicate.

^b^K_i_ value of Sol g 2.1 protein and various ligands investigated by fluorescence competitive binding assay.

**Fig. 3:**
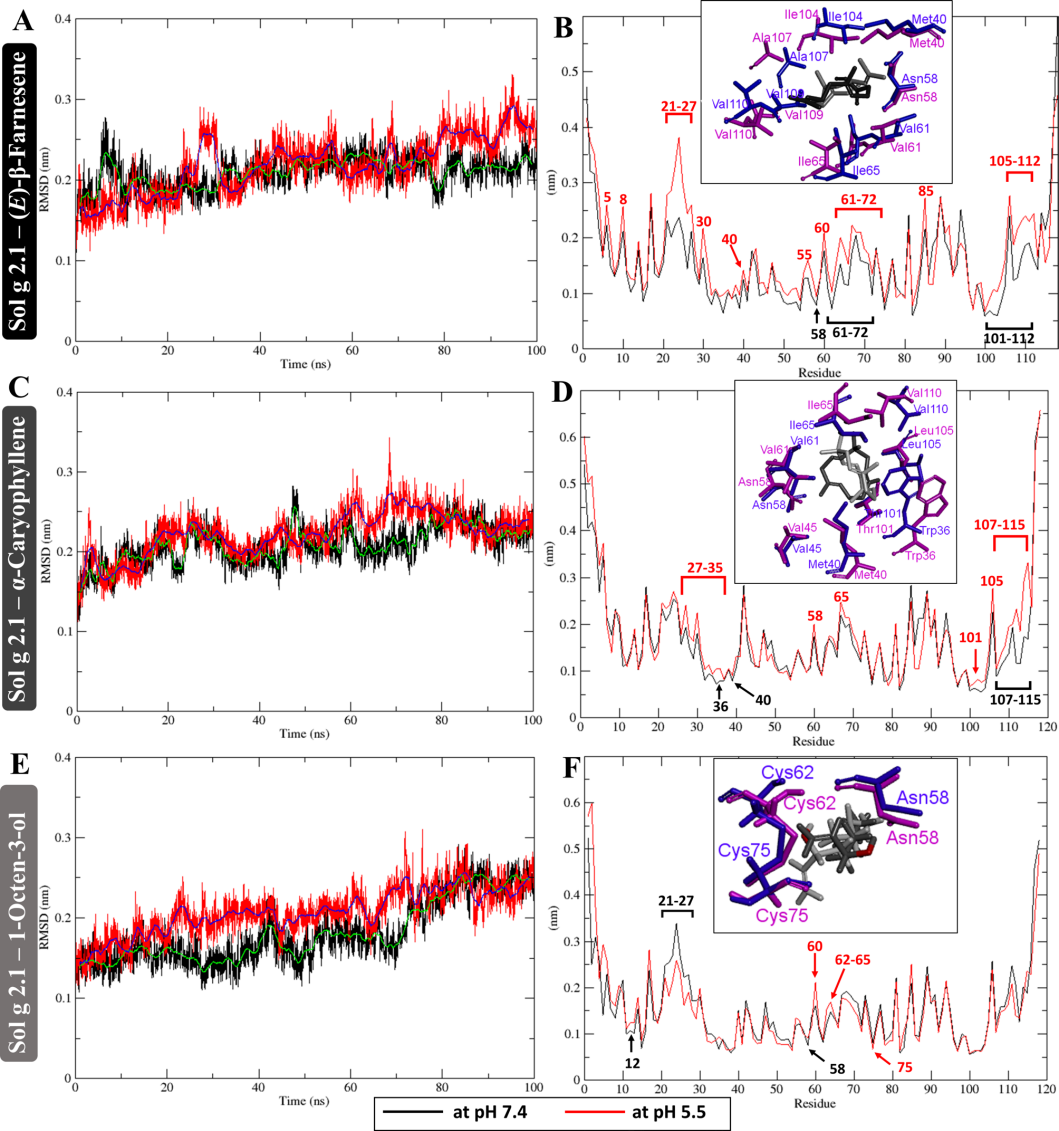
(**A**), (**C**), and (**E**) Show backbone RMSD of the Sol g 2.1 protein and (*E*)-β-Farnesene, α-Caryophyllene, and 1-Octen-3-ol, respectively. (**B**), (**D**), and (**E**) showed RMSF of all residues Sol g 2.1 protein after simulation with the ligands including, (*E*)-β-Farnesene, α-Caryophyllene, and 1-Octen-3-ol, respectively. Red and black lines represent at pH 5.5 and 7.4. Insert boxes compared fluctuation amino acid residues and ligands at pH 7.4 and 5.5. Blue and magenta indicated amino acids of Sol g 2.1 at pH 7.4 and 5.5, respectively. Dark gray and pale gray represented ligands at pH 7.4 and 5.5, respectively.

## Molecular docking

The top rank of docking score with the lowest RMSD of Sol g 2.1 (GenBank: UYX46120.1) with (*E*)-β-Farnesene, α-Caryophyllene, and 1-Octen-3-ol complexes were selected for molecular dynamics simulation (as shown in Figure S4). All of the selected poses have the RMSD lower than 2 Å (Table 1)^27^. At the binding site after the receptor was protonated at pH 7.4 and 5.5, PLB (propensity for ligand binding) were 0.75 and 1.52, respectively which were the most suitable binding sites between the protein and ligands. (*E*)-β-Farnesene and α-Caryophyllene were surrounded by mostly non-polar amino acids, including Trp36, Met40, Val61, Ile65, Ile66, Ile79, Ile104, Val109, and Val110 of Sol g 2.1. Extraordinary, there was Ile100 amino acid, which contacts with (*E*)-β-Farnesene. However, 1-octen-3-ol ligand, there were fewer non-polar amino acids surrounded, including Trp36, Val61, Ile65, Ile79, Ile104, Val109, and Val110. Interestingly, Thr113 and Tyr46 formed hydrogen-bonded the OH group to 1-Octen-3-ol. These results revealed that (*E*)-β-Farnesene and α-Caryophyllene were better ligands for Sol g 2.1 than 1-Octen-3-ol. This is reasonable considering the highly hydrophobic nature of the pocket interior of the protein preferring to bind hydrophobic ligands^4,28^.

## Molecular dynamics analysis of the stability

After 100 ns simulations, the final simulation structures of Sol g 2.1 with (*E*)-β-Farnesene, α-Caryophyllene, and 1-Octen-3-ol at pH 7.4 (deep blue ribbon) and pH 5.5 (magenta ribbon) were shown in Fig. 2A, B and C, respectively. The final simulated structures were superposed with initial simulation (gray ribbon) for examination of the protein conformation changes^29^. There was an obvious difference in the helical loop unwinding at pH 5.5 and fluctuated loop regions between the two structures (pH 7.4 and pH 5.5), especially N- and C-termini loops, as indicated by the black boxes (as shown in Fig. 2).

An average RMSD backbone of Sol g 2.1‒(*E*)-β-Farnesene at pH 7.4 and 5.5 were 2.09 Å and 2.20 Å, respectively (as shown in Fig. 3A). The value of RMS distribution in pH 7.4 and 5.5 fluctuated in ranges 3.0 Å (pH 7.4) and 3.3 Å (pH 5.5), respectively. There were highly fluctuates at position α1 (residues 5 and 8), β-loop 21–27, α1 (residue 30 and 40), α3 (residues 55 and 60–72), α4 (residue 85), and α5 (105–112) in pH 5.5. However, there were low fluctuations at residues 58, 61–72, and 101–112 in pH 7.4. These are the inner hydrophobic cavity binding. This means that (*E*)-β-Farnesene is more stable in the inner cavity of Sol g 2.1 at pH 7.4 than at pH 5.5 (as shown in Fig. 3B). Next, Sol g 2.1‒α-Caryophyllene, an average RMSD of the backbone at pH 7.4 and 5.5 were 2.08 Å and 2.22 Å, respectively. The backbones have varied in ranges of 3.1 Å (pH 7.4) and 3.5 Å (pH 5.5) (as shown in Fig. 3C). At pH 5.5, at position α1 (residues 27–35), α3 (residues 58 and 65), and α5 (residues 101, 105, and 107–115) had high fluctuation. However, at pH 7.4, there were amino acids at positions 36, 40, and 107–115 showing low fluctuation. This result revealed that Sol g 2.1 at pH 7.4 is suitable for α-Caryophyllene binding when compared with the lower pH condition (as shown in Fig. 3D). In the complex of Sol g 2.1‒1-Octen-3-ol, the backbones at pH 7.4 and 5.5 have an average RMSD, including 1.81 Å and 2.07 Å, respectively. The backbones have 3.0 Å (pH 7.4) and 3.1 Å (pH 5.5) varied ranges of fluctuation (as shown in Fig. 3E). There were high fluctuations of residues 60, and 62–65 in the protein backbone at pH 5.5 than at pH 7.4. Importantly, the amino acids at position 58 (at pH 7.4) and 75 (at pH 5.5) play key amino acid residues (as shown in Fig. 3F)^30^. Thus, we hypothesized that these amino acid residues have a strong relationship in the formation of interaction with compounds.

**Fig. 4:**
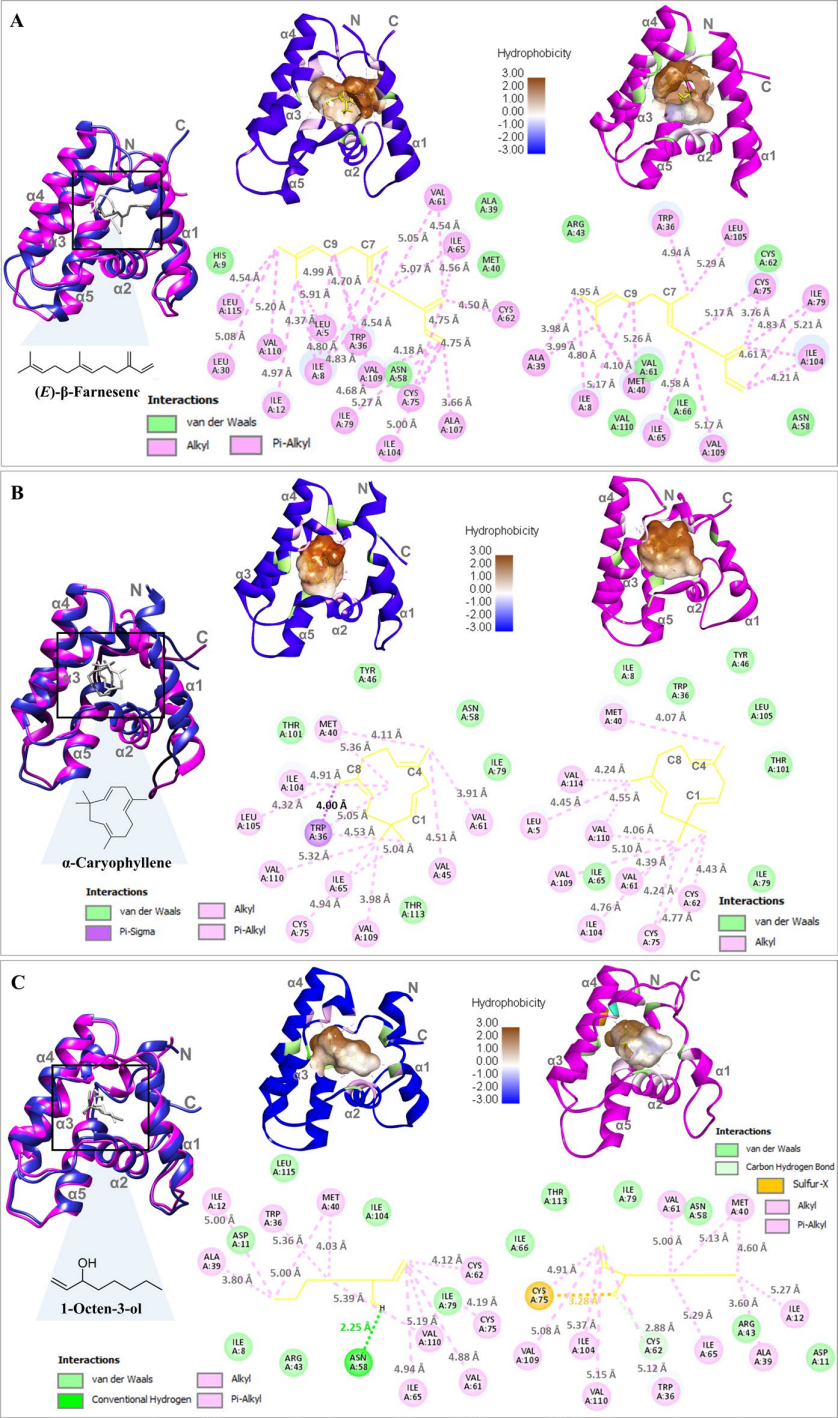
(**A**‒**C**) Superimposed of Sol g 2.1 complexes with Sol g 2.1 with (*E*)-β-Farnesene, α-Caryophyllene, and 1-Octen-3-ol α after 100 ns simulation at pH 7.4 (blue ribbon, dark gray stick) and 5.5 (magenta ribbon, pale gray stick), respectively. In the gray boxes, the top section depicts a 3D schematic diagram showing the conformations and the residues of Sol g 2.1 that interact with the ligands (yellow sticks). The brown surface highlights the hydrophobic region within the inner pocket. The bottom section presents a 2D interaction analysis of Sol g 2.1 with (E)-β-Farnesene (A), α-Caryophyllene (B), and 1- Octen-3-ol (C).

As mentioned above, the complexes of Sol g 2.1 with (*E*)-β-Farnesene and α-Caryophyllene at pH 7.4, residues 61–72 and 101–112 have low fluctuations as well as they were inner cavity regions of Sol g 2.1 protein (α3-α5 regions). Thus, these areas play a crucial role in binding and stabilizing ligands. From Sol g 2.1‒(*E*)-β-Farnesene, results revealed that the ligand was stabilized in the inner hydrophobic pocket by Alkyl and Pi-Alkyl interactions. Moreover, Trp36, which is a key amino acid interacted with a methyl group at the C7-position of (*E*)-β-Farnesene with 4.54 Å. It was a shorter distance at pH 5.5 with 4.94 Å (as shown in Fig. 4A). This result concluded that Sol g 2.1 was stable for (*E*)-β-Farnesene binding at pH 7.4. Next, in the complexes of Sol g 2.1 with α-Caryophyllene, more amino acids were interfacing with the ligands at pH 7.4 than at pH 5.5. Importantly, Trp36 did Pi-Sigma interaction with the ligand at the C8-methyl group. Thus, α-Caryophyllene ligand was suitable for binding in Sol g 2.1 pocket in the physiological environment (as shown in Fig. 4B). Further, in the complexes of Sol g 2.1 with 1-Octen-3-ol, there was a key H-bond between the hydroxyl group of 1-Octen-3-ol and N58 receptor at pH 7.4. In addition, Met40 which was a key amino acid interacted with the same position of this ligand at both pHs. It did alkyl interaction with C5 of this ligand with 4.03 Å and 5.13 Å, and C8 with 5.00 Å and 4.60 Å at pH 7.4 and 5.5, respectively. Moreover, 1-Octen-3-ol has a closer distance with the inner receptor residues at pH 7.4 than at pH 5.5. Nevertheless, at pH 5.5, there was Sulfer-X between the hydroxyl group of the ligand and Cys75 of the receptor. Moreover, the carbon-hydrogen bond interaction of Cys62 and the hydroxyl group 1-Octen-3-ol (as shown in Fig. 4C). These results demonstrated that the structure of Sol g 2.1 at pH 7.4 closely resembles the crystal structure and exhibits stronger and more stable binding with ligands compared to the structures at pH 5.5 (Table 2).

**Table 2:** Molecular dynamics simulation results of particular ligands.

	Ligand	H-Bond and Sulfer-X	Hydrophobic interactions (Alkyl, Pi-Alkyl, Sigma-Alkyl)	Van der-Waals
pH 7.4	(*E*)-β-Farnesene	–	LEU5, ILE8, ILE12, LEU30, TRP36, VAL61, CYS62, ILE65, CYS75, ILE79, ILE104, ALA107, VAL109, VAL110, LEU115	HIS9, ALA39, MET40, ASN58
	α-Caryophyllene	–	TRP36, MET40, VAL45, VAL61, ILE65, CYS75, ILE104, LEU105, VAL109, VAL110	TYP46, ASN58, ILE79, THR101, THR113
	1-Octen-3-ol	ASN58 (H-Bond)	ILE12, TRP36, ALA39, MET40, , VAL61, CYS62, ILE65, CYS75, VAL110	ILE8, ASP11, ARG43, ILE79, ILE104, LEU115
pH 5.5	(*E*)-β-Farnesene	–	ILE8, TRP36, ALA39, MET40, ILE65, CYS75, ILE79, ILE104, LEU105, VAL109	ARG43, ASN58,VAL61, CYS62, ILE66, VAL110
	α-Caryophyllene	–	LEU5, MET40, VAL61, CYS62, CYS75, ILE104, VAL109, VAL110, VAL114	ILE8, TRP36, TYP46, ILE65, ILE79, THR101, LEU105
	1-Octen-3-ol	CYS62, CYS75 (Sulfer-X)	ILE12, TRP36, ALA39, MET40, , VAL61, ILE65, ILE104, VAL109, VAL110	ASP11, ARG43, ASN58, ILE66, ILE79, THP113

**Fig. 5:**
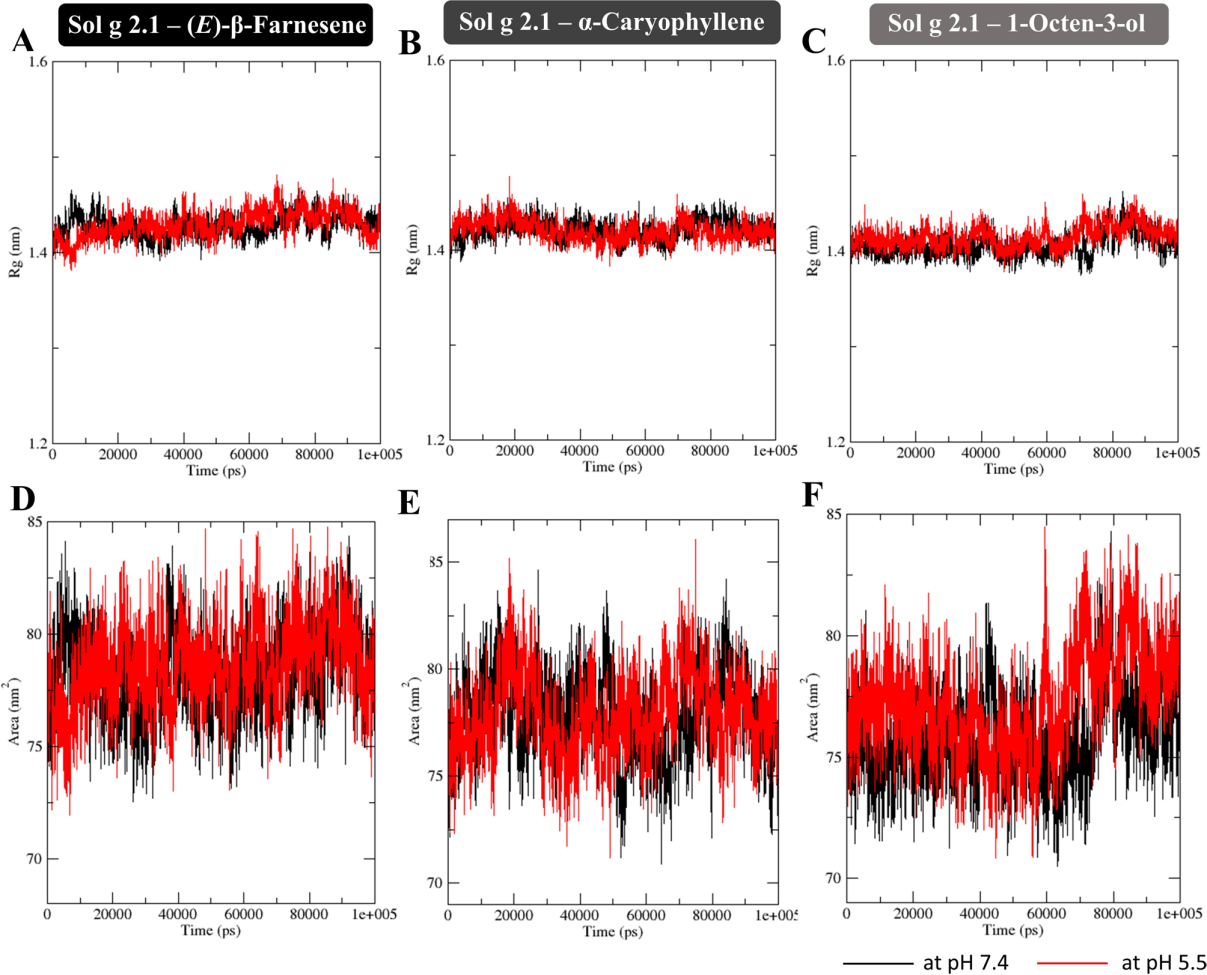
(**A**‒**C**) Time evolution of radius of gyration (Rg) of Sol g 2.1 protein and (*E*)-β-Farnesene, α-Caryophyllene, and 1-Octen-3-ol complexes, respectively. (**D**‒**E**) Solvent Accessible Surface Area (SASA) of Sol g 2.1 protein and (*E*)-β-Farnesene, α-Caryophyllene, and 1-Octen-3-ol complexes, respectively. The Black and red color represent the protein and ligands complexes at pH 7.4 and 5.5, respectively.

The radius of gyration (Rg) was conducted to measure the compactness of a protein, indicating how spread out the atoms are from the center of mass. Stability in this measure can indicate that the protein structure has reached equilibrium^31^. For Sol g 2.1‒(*E*)-β-Farnesene simulation, in both conditions (at pH 7.4 and 5.5), after 100 ns, the Rg value appeared to have reached a stable value with smaller fluctuations compared to the earlier parts of the simulation. This means that the protein structure likely reached equilibrium at nearly 100 ns. The average Rg values of Sol g 2.1 complexes with (*E*)-β-Farnesene, α-Caryophyllene, and 1-Octen-3-ol at pH 7.4 were 1.42 nm, 1.42 nm, and 1.40 nm, respectively. At pH 5.5, the values were 1.43 nm, 1.42 nm, and 1.41 nm, respectively. In included, we observed larger (Sol g 2.1 complexes with (*E*)-β-Farnesene and α-Caryophyllene) fluctuations and irregular (Sol g 2.1 complexes with 1-Octen-3-ol) fluctuations at the lower pH, while the protein complexes at pH 7.4 appeared more stable and consistent over time. This suggests that the protein structure has reached a more stable equilibrium at the physiological pH (as shown in Fig. 5A‒C).

The average Solvent Accessible Surface Area (SASA) values of the protein complex with (*E*)-β-Farnesene, α-Caryophyllene, and 1-Octen-3-ol at pH 7.4 were 77.34 nm^2^, 77.08 nm^2^, and 75.80 nm^2^, respectively. At pH 5.5, the average SASA values of the Sol g 2.1 protein with these ligands were 78.63 nm^2^, 77.98 nm^2^, and 77.29 nm^2^, respectively (as shown in Fig. 5D‒F). Since ligand binding involves solvent replacement, lower SASA values indicate that the binding pocket is less exposed to solvent, suggesting that the ligand remains within the binding pocket during the simulation^32^. Thus, the Sol g 2.1 complexes with (*E*)-β-Farnesene, α-Caryophyllene, and 1-Octen-3-ol at pH 7.4 exhibited minor structural and solvent accessibility changes, indicating that the protein and ligand structure has reached a more stable equilibrium^33^.

## Structural analysis of pH-induced changes

It is well-known that even a minor change in the protonation state of a titrating residue can significantly alter protein conformation, leading to different ligand binding poses^28^. After 100 ns simulation at pH 7.4 and 5.5, we found that the protein complexes had more negatively charged residues (Asp and Glu), some partially protonated residues (His), and positively charged residues (Lys and Arg), leading to a mixed charge distribution at pH 7.4 (Table 3). Importantly, ASP34 and GLU4 exhibit significant pKa shifts, indicating they may play crucial roles in protonation and conformational changes. Additionally, CYS62 and CYS75 had high pKa values, suggesting they were likely involved in disulfide bond formation and stability. Furthermore, at pH 5.5, Sol g 2.1 complexes with (*E*)-β-Farnesene, and α-Caryophyllene had more total average protonation for basic residues, including 3.52 (3.40 at pH 7.4) and 3.63 (3.58 ay pH 7.4), respectively. Possibly, these complexes had an overall positive charge at the lower pH (Table 4). The higher total molecular protonation at pH 5.5 could result in some significant conformational changes of Sol g 2.1 protein complexes^34^. This may prove that the protein in pH 5.5 undergoes a larger conformational shift and is less stable^11^.

**Table 3:** The pKa predictions and protonation fractions for acidic and basic residues in Sol g 2.1 protein complexes with (*E*)-β-Farnesene, α-Caryophyllene, and 1-Octen-3-ol were calculated from a 100 ns simulation at pH 7.4 and pH 5.5. Theoretical pKa values predicted by PDB2PQR software are represented as pKa values.

Amino acids	pKa prediction of Sol g 2.1 with (*E*)-β-Farnesene		pKa prediction of Sol g 2.1 with α-Caryophyllene		pKa prediction of Sol g 2.1 with 1-Octen-3-ol		pK model
	pH 7.4	pH 5.5	pH 7.4	pH 5.5	pH 7.4	pH 5.5	
ASP11	3.63	3.28	3.27	2.96	3.87	2.77	3.80
ASP27	3.74	3.66	3.55	3.21	3.41	3.47	3.80
ASP28	3.33	2.57	2.39	2.51	4.01	2.96	3.80
ASP34	1.16	0.67	0.50	2.56	1.02	1.04	3.80
ASP47	3.94	4.08	3.97	3.87	3.87	4.00	3.80
ASP68	2.00	3.00	3.03	3.07	3.28	2.81	3.80
ASP84	3.21	3.80	3.28	3.87	3.94	3.87	3.80
GLU3	3.91	4.50	4.57	4.52	4.50	3.98	4.50
GLU4	1.65	4.50	4.50	3.77	3.91	2.31	4.50
GLU56	4.50	4.50	4.50	4.50	4.50	4.50	4.50
GLU73	4.50	3.84	4.05	3.91	3.91	4.57	4.50
GLU86	4.10	3.40	4.50	3.92	4.26	4.07	4.50
GLU111	4.50	3.94	4.38	4.57	3.50	4.50	4.50
HIS1	6.43	7.24	6.36	6.50	6.36	6.50	6.50
HIS9	6.50	6.43	6.52	6.50	7.06	6.50	6.50
HIS37	5.32	4.02	4.14	5.20	3.87	4.60	6.50
CYS15	9.21	8.94	8.83	8.44	> 12	> 12	9.00
CYS22	8.31	8.99	8.87	8.23	8.68	9.00	9.00
CYS38	9.07	8.06	9.07	8.08	> 12	> 12	9.00
CYS62	> 12	> 12	> 12	> 12	> 12	> 12	9.00
CYS75	> 12	> 12	> 12	> 12	> 12	> 12	9.00
CYS82	> 12	> 12	> 12	> 12	> 12	> 12	9.00
CYS103	> 12	> 12	> 12	> 12	> 12	> 12	9.00
TYR46	10.44	10.74	10.61	11.08	10.70	10.63	10.00
LYS10	10.36	10.50	10.29	10.43	10.29	10.50	10.50
LYS14	10.50	10.50	10.50	10.50	10.50	10.50	10.50
LYS21	10.50	10.50	10.43	9.94	10.50	10.50	10.50
LYS42	10.22	10.43	10.29	10.22	10.50	10.50	10.50
LYS55	10.15	10.36	10.50	10.22	10.36	10.01	10.50
LYS57	10.29	10.15	10.01	10.22	10.43	10.43	10.50
LYS60	10.50	10.36	10.50	10.22	10.43	10.50	10.50
LYS64	10.15	10.36	10.36	10.08	10.29	10.29	10.50
LYS76	10.29	10.50	10.15	10.29	10.29	10.29	10.50
LYS77	10.50	10.22	10.50	10.50	10.50	10.36	10.50
LYS96	10.36	10.36	9.80	10.15	10.08	9.73	10.50
LYS118	10.50	10.22	10.36	10.50	10.50	10.50	10.50
ARG17	12.50	12.22	12.36	11.94	11.80	12.50	12.50
ARG32	11.24	11.66	11.52	11.38	11.87	11.73	12.50
ARG43	12.08	11.92	12.43	12.08	11.43	11.67	12.50
ARG81	12.15	12.22	12.15	12.15	12.22	12.50	12.50
ARG85	12.36	12.15	11.94	12.15	12.29	12.36	12.50
ARG89	12.22	12.50	12.50	12.08	12.50	12.36	12.50
ARG91	11.24	11.59	12.08	11.24	12.43	12.01	12.50
ARG94	12.50	12.22	12.08	12.50	12.22	12.50	12.50
ARG106	12.50	12.50	12.36	12.36	12.50	12.15	12.50
ARG117	11.94	11.94	12.29	12.15	12.01	12.01	12.50

**Table 4:** The total average of molecular protonation for the acidic, basic and neutral amino acids groups of Sol g 2.1 protein complexes with (*E*)-β-Farnesene, α-Caryophyllene, and 1-Octen-3-ol pH 7.4 and pH 5.5 after 100 ns simulation.

Group	pKa prediction of Sol g 2.1 with (*E*)-β-Farnesene		pKa prediction of Sol g 2.1 with α-Caryophyllene		pKa prediction of Sol g 2.1 with 1-Octen-3-ol	
	pH 7.4	pH 5.5	pH 7.4	pH 5.5	pH 7.4	pH 5.5
Acidic	3.40	3.52	3.58	3.63	3.69	3.45
Basic	10.53	10.52	10.50	10.47	10.52	10.54
Neutral	10.63	10.59	10.67	10.48	10.67	11.45

## Binding free energy calculation

The binding free energy (∆*G*_*bind*_) was calculated by summing the binding free energy (∆*G*_*MM-PBSA*_) obtained from the Molecular Mechanics Poisson-Boltzmann Surface Area method and the interaction entropy derived from the Interaction Entropy (IE) method. The IE method was employed to calculate the entropy contribution, offering greater efficiency and theoretical rigor compared to the normal mode method^35^^‒^^37^. Table 5 presents a summary of the binding free energy based on all MD simulation trajectories. The results demonstrated that both methods showed greater stability with lower binding free energy for the Sol g 2.1 protein and all ligand complexes at pH 7.4 compared to pH 5.5. The ∆*G*_*bind*_ values of the Sol g 2.1 complexes with (*E*)-β-Farnesene, α-Caryophyllene, and 1-Octen-3-ol at pH 7.4 were − 48.74 kcal/mol, − 49.29 kcal/mol, and − 8.45 kcal/mol, respectively. These values indicate lower binding free energy at physiological pH compared to pH 5.5, suggesting that the Sol g 2.1 protein has a reduced affinity for these ligands at the lower pH.

**Table 5:** The calculation of binding free energy (∆*G*_*bind*_) of the complexes of Sol g 2.1 protein and (*E*)-β-Farnesene, α-Caryophyllene, and 1-Octen-3-ol at pH 7.4 and 5.5.

	Ligand	∆*G*_*gas*_	∆*G*_*sol*_	∆*G*_*MM-PBSA*_	‒*T∆S*	∆*G*_*bind*_
pH 7.4	(*E*)-β-Farnesene	‒37.29	7.58	‒29.71	78.45	‒48.74
	α-Caryophyllene	‒36.71	7.31	‒29.40	78.69	‒49.29
	1-Octen-3-ol	‒25.99	7.45	‒18.54	26.99	‒8.45
pH 5.5	(*E*)-β-Farnesene	‒36.01	6.39	‒29.62	75.93	‒46.31
	α-Caryophyllene	‒36.67	7.55	‒29.12	74.21	‒45.09
	1-Octen-3-ol	‒24.79	8.24	‒16.55	23.14	‒6.59

All energies are in kcal/mol. Total gas phase energy (∆*G*_*gas*_) consists of the electrostatic energy (∆*G*_*ele*_) and the van der Waals energy (∆*G*_*vdW*_) that are significant for binding. Total solvation energy (∆*G*_*sol*_) consists of non-polar solvation energy (∆*G*_*np*_) and polar solvation energy (∆*G*_*pb*_). Binding free energy (∆*G*_*MM-PBSA*_) was calculated from the terms above (∆*G*_*gas*_ + ∆*G*_*sol*_). Δ*G*_*bind*_ is binding free energy that was obtained from summing up ∆*G*_*MM-PBSA*_ and *–TΔS.*

## Residue-based decomposition

**Fig. 6:**
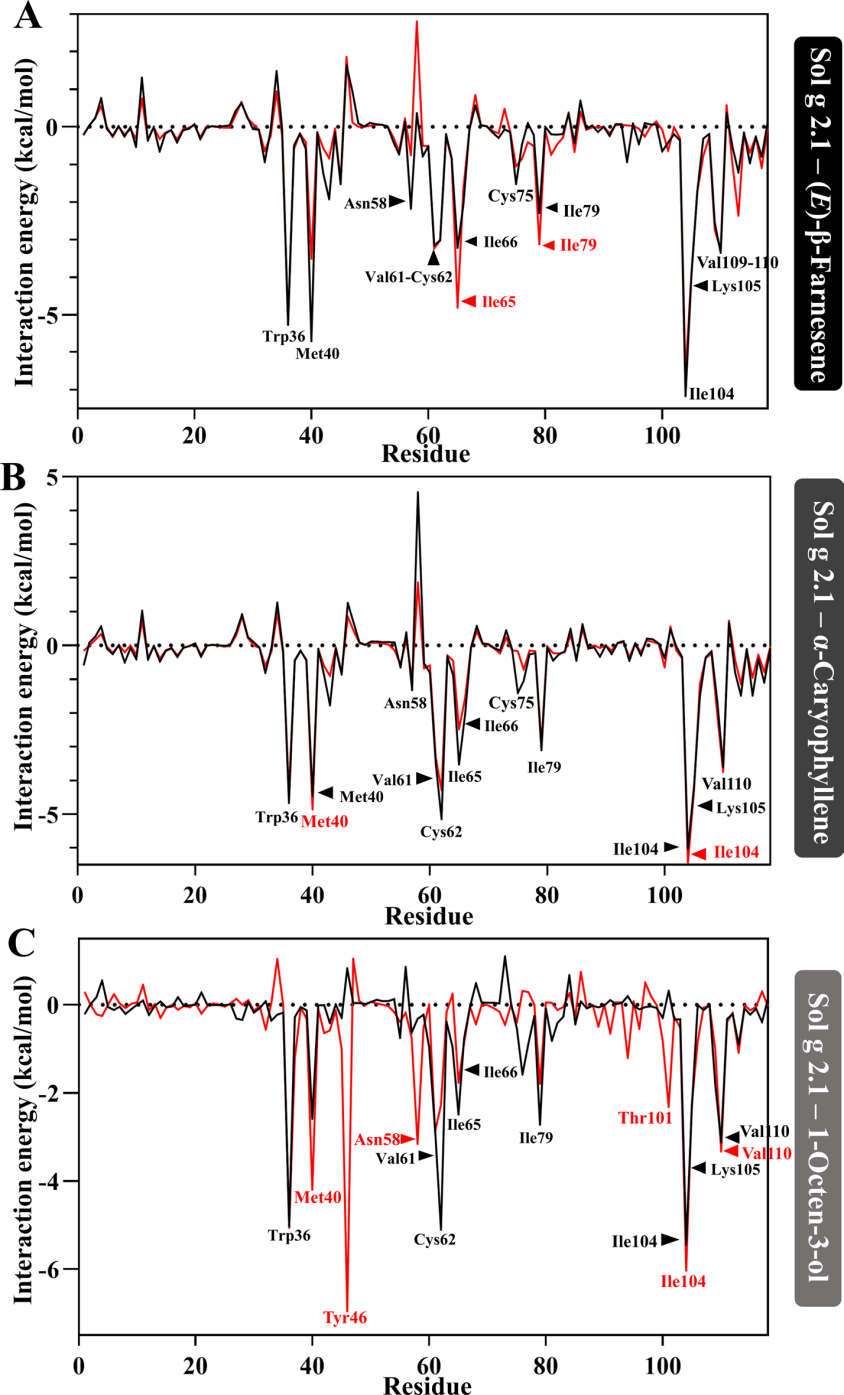
(**A**), (**B**), and (**C**) The interaction energy (kcal/mol) of per-residue composition was analyzed over the production period for the Sol g 2.1 protein complexes with (*E*)-β-Farnesene, α-Caryophyllene, and 1-Octen-3-ol, respectively. The interactions were represented in black for the protein–ligand complexes at pH 7.4 and in red for those at pH 5.5.

The energy decomposition of Sol g 2.1 protein and ligand complexes was conducted to analyze the individual contributions of each residue in the active site^38^. The interactions were examined to assess the stability of the complex based on these residues. In the Sol g 2.1 protein complexes with (*E*)-β-Farnesene, α-Caryophyllene, and 1-Octen-3-ol at pH 7.4, we found that the major contributors to the binding free energy were the residues at the active site, including Trp36, Val61, Cys62, Ile66, and Lys105. Additionally, Met40 was a major contributor in the Sol g 2.1 protein and (*E*)-β-Farnesene complex. There were also residues Asn58, Cys75, and Val110 in the complexes with (*E*)-β-Farnesene and α-Caryophyllene, and residues Ile65 and Ile79 in the complexes with α-Caryophyllene and 1-Octen-3-ol, which showed lower interaction energy than in an acidic pH environment (as shown in Fig. 6A, B). In comparison to the Sol g 2.1 protein and ligand complexes at lower pH, there were a few residues that acted as major contributors, including Met40 and Ile104 in the complexes with α-Caryophyllene and 1-Octen-3-ol. Moreover, Ile65 and Ile79 were major contributors in the complex with (*E*)-β-Farnesene, and Thr101 was a major contributor in the complex with 1-Octen-3-ol (as shown in Fig. 6).

## Principal Component Analysis

**Fig. 7:**
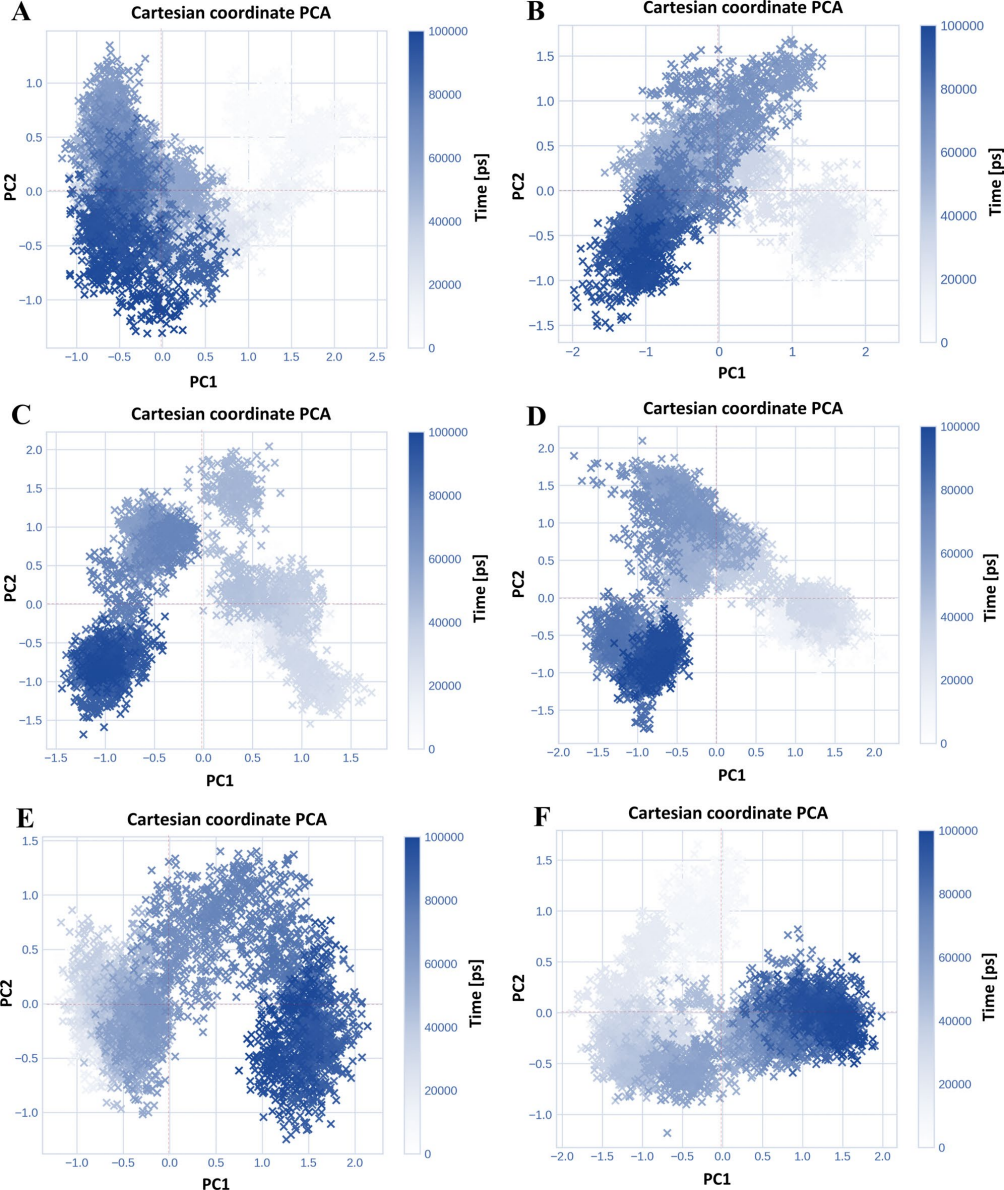
Virtualizing of Cartesian coordinate conformation changes over the course of the simulation of the Sol g 2.1 protein and its ligands complexes. The distribution of white and blue crosses “x” illustrates the extent of conformational changes during the simulation (100 ns). The color scale, ranging from white to blue, corresponds to the simulation time, with white indicating the initial time step, and blue representing the final time stages. (**A**), (**C**), and (**E**) The complexes of Sol g 2.1 with (*E*)-β-Farnesene, α-Caryophyllene, and 1-Octen-3-ol at pH 7.4, respectively. (**B**), (**D**), and (**F**) The complexes of Sol g 2.1 with (*E*)-β-Farnesene, α-Caryophyllene, and 1-Octen-3-ol at pH 5.5, respectively.

Cartesian coordinate principal component analysis (PCA) of the molecular dynamics simulation trajectories presented illustrating the findings of a study on the random global movement of atoms in amino acid residues of Sol g 2.1 protein complexes. At pH 7.4, the conformational cluster points of the Sol g 2.1 protein and its complexes with (*E*)-β-Farnesene, α-Caryophyllene, and 1-Octen-3-ol were more distinct and separated, particularly in the darker regions shown in Fig. 7A, C, and D, respectively. Specifically, the Sol g 2.1 and (*E*)-β-Farnesene complex points at pH 7.4 were densely packed in a particular region, indicating that the protein complex conformations were more localized within a specific area of the PCA space (as shown in Fig. 7A). The principal component ranges of PC1 for the complexes with (*E*)-β-Farnesene, α-Caryophyllene, and 1-Octen-3-ol were ‒1.0 to 2.5, ‒1.5 to 1.5, and ‒1.0 to 2.0, respectively. Additionally, the PC2 ranges for these complexes were ‒1.0 to 1.0, ‒1.5 to 2.0, and ‒1.0 to 1.5, respectively. At pH 5.5, the distribution of points for the Sol g 2.1 protein and (*E*)-β-Farnesene showed a broader spread, suggesting a wider range of conformations explored during the simulation (as shown in Fig. 7B). The points formed a more continuous spread, with noticeable clustering, particularly in the darker regions (as shown in Fig. 7B, E, and F). The principal component ranges of PC1 for the complexes with (*E*)-β-Farnesene, α-Caryophyllene, and 1-Octen-3-ol were ‒2.0 to 2.0. The PC2 ranges for these complexes were ‒1.5 to 1.5, ‒1.5 to 2.0, and ‒1.0 to 1.5, respectively. In conclusion, the graphs for Sol g 2.1 protein and its ligand complexes at pH 7.4 displayed more localized and denser clustering, indicating that these protein complexes remain in specific conformational states for longer periods, suggesting higher stability. In contrast, at pH 5.5, the broader spread of points and less distinct clustering suggested that the protein explores a wider range of conformational states and transitions between them more frequently^39^.

## Discussion

The Sol g 2.1 protein exhibited significant similarity to Sol i 2 and demonstrated the ability to bind various potential ligands^4^. Moreover, prior research suggested that under acidic pH conditions, the tertiary structure of the Sol g 2.1 protein becomes less rigid, potentially enabling it to function as a carrier for active molecules, delivering them to target cells via pH gradients^3^. Furthermore, the Sol g 2.1 protein showed a strong affinity for binding hydrophobic ligands, including analogs of fire ant trails pheromones and piperidine sidechains^40^. Based on this, it is plausible to hypothesize that the protein could facilitate the transportation of hydrophobic compounds, binding to receptors, and subsequently releasing the ligands on neuronal cell membranes or other cell targets such as cancer cells with an acidic environment^3,31,41^.

In this study, we first investigated the binding characteristics of Sol g 2.1 protein with potential ligands, including (*E*)-β-Farnesene, α-Caryophyllene, and 1-Octen-3-ol, through a series of experimental and computational approaches. The fluorescence competitive binding assay provided insights into the affinity binding between Sol g 2.1 protein and the ligands. We determined the equilibrium dissociation constant (K_d_) of Sol g 2.1 protein with 1-NPN at different pH levels, including pH 5.5 and 7.4^42^. Our results indicated a higher affinity binding of Sol g 2.1 protein with 1-NPN at pH 7.4 compared to pH 5.5^43^. Furthermore, we evaluated the binding affinities of Sol g 2.1 protein with the competitive ligands, revealing varying degrees of affinity at different pH levels. Notably, (*E*)-β-Farnesene exhibited the strongest binding affinity with Sol g 2.1 protein at pH 7.4, followed by α-Caryophyllene and 1-Octen-3-ol^4^. However, at pH 5.5, the binding preferences shifted, indicating pH-dependent binding characteristics of Sol g 2.1 protein with the ligands. From these results, we concluded that Sol g 2.1 protein has a suitable structure for binding with the various ligands at the physiological pH, while it has lower binding ability with the ligands at the acidic pH. Thus, Sol g 2.1 protein acts as a pH-sensitive carrier protein, which delivers in response to the pH difference between normal and cancer cells^44^.

Molecular docking simulations provided structural insights into the interactions between Sol g 2.1 protein and the ligands. The simulations revealed key amino acid residues involved in ligand binding and highlighted the hydrophobic nature of the ligand-binding pocket of the Sol g 2.1 protein model. Notably, (*E*)-β-Farnesene and α-Caryophyllene were identified as better ligands for Sol g 2.1 protein compared to 1-Octen-3-ol at pH 7.4, indicating by S score with the lowest RMSD of the complexes^45^. However, at pH 5.5, all ligands showed lower affinity binding with Sol g 2.1 protein, owing to their favorable interactions with the hydrophobic pocket^46^. Interestingly, at pH 7.4 and pH 5.5, the orientation and positioning of the molecules in relation to other residues (such as Val, Ile, and Trp) were different, indicating that the interactions between these residues and the compounds were pH-dependent. The changes in orientation suggested that the interactions between the compound and its surrounding residues were optimized differently at each pH level (as shown in Fig. S4). Molecular dynamics simulations further elucidated the dynamic behavior of Sol g 2.1 protein–ligand complexes at both pH conditions.

The simulations revealed conformational changes in Sol g 2.1 protein upon ligand binding and provided insights into the stability of the complexes. Interestingly, Sol g 2.1 protein exhibited higher stability and stronger binding with the ligands at pH 7.4 compared to pH 5.5 during 100 ns simulation. Interestingly, Sol g 2.1 demonstrated stronger and more stable binding with all three ligands at pH 7.4 compared to pH 5.5. To investigate the roles of key residues, non-polar amino acids at the active site were identified as crucial for the interaction between the Sol g 2.1 protein and the ligands, as they exhibited low fluctuations, particularly at neutral pH (as shown in Fig. 3). From the radius of gyration analysis revealed that at pH 5.5, the Rg values were slightly higher, indicating more fluctuations and less stability compared to pH 7.4. In addition, SASA values implied that the ligand remained more securely within the binding pocket at the physiological pH of 7.4, leading to a more stable equilibrium state. the results suggest that the protein and ligand complexes are more stable and consistent at pH 7.4, indicating a more stable equilibrium state under physiological conditions^32,33^. The protein protonate prediction revealed that ASP34 and GLU4 have significant pKa shifts indicating potential roles in protonation and conformational changes. Moreover, CYS62 and CYS75 had high pKa values suggesting involvement in disulfide bond formation and stability. These findings highlighted the importance of pH in modulating the stability and binding efficiency of protein–ligand complexes. Importantly, further mutating these can reveal their specific roles in ligand binding and stability^47^.

The binding free energy analysis ∆*G*_*bind*_ indicated that the total binding free energy was negative for all complexes, signifying favorable binding interactions. Notably, the Sol g 2.1 protein and all ligand complexes exhibited lower energy at physiological pH compared to acidic pH. However, the ∆*G*_*vdW*_ values of the complexes at pH 7.4 were not significantly lower than at pH 5.5. This suggests that the ligand binding pocket, which contains several non-polar amino acids, continues to influence ligand binding under acidic conditions^48^. Moreover, these findings were related to the fluorescence competitive binding assay. In terms of per-residue composition, several active site residues were identified as major contributors (e.g., Trp36, Met40, Val61, Cys62, Ile66, Lys105, etc.). These residues play critical roles in stabilizing the Sol g 2.1 protein–ligand complexes at physiological pH. Their contributions to the binding free energy help maintain the structural integrity and functional interactions within the active site. However, some residues assist in maintaining the structural interactions of the protein complexes at acidic pH. Thus, the varying contributions of specific residues at different pH levels highlight the dynamic nature of protein–ligand interactions. Protonation states influenced by pH can significantly impact the binding free energy and, consequently, the stability of the complex^49^.

**Fig. 8:**
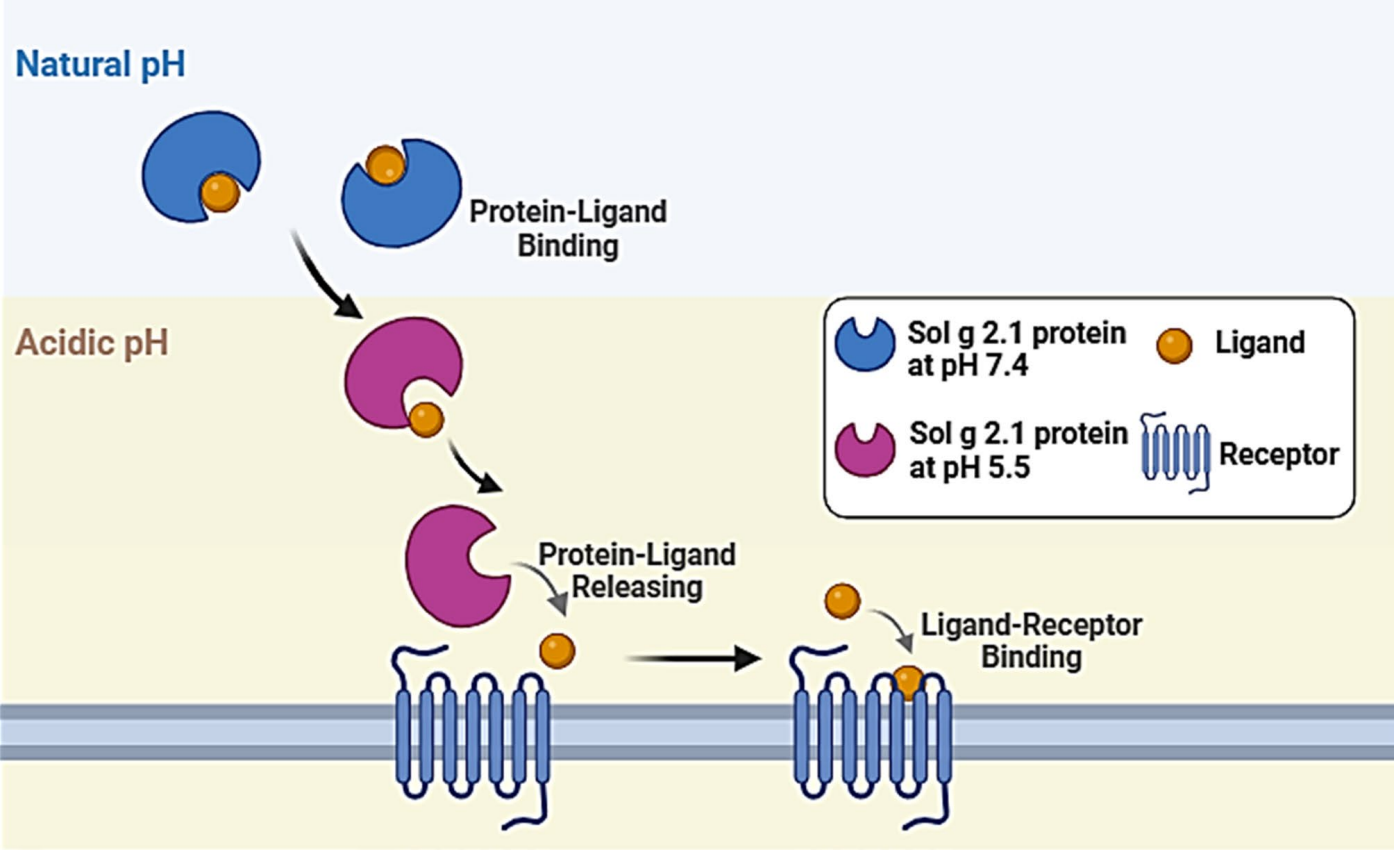
Schematic of Sol g 2.1 protein–ligand binding forms in different pH environments.

From PCA results, we concluded that at pH 7.4, the Sol g 2.1 protein and its ligand complexes exhibited tightly packed and localized clusters. This implies that these complexes persisted in specific conformational states for extended periods, indicating greater stability. In contrast, at pH 5.5, the wider distribution and less defined clustering suggested that the protein sampled a broader array of conformational states and transitions between them more frequently^33^. Therefore, we suggested that Sol g 2.1 protein is pH-responsive, leading to conformational binding pocket changes and losing binding capacity with the ligands at the acidic pH^11,47,49–51^. Considering the presence of the various ligands at the physiological pH of Sol g 2.1 protein, it would be intriguing to investigate whether the acidic extracellular microenvironment in cancer cells contributes to the modified ligand preference of the protein^50^ (as shown in Fig. 8). Therefore, we hypothesized that Sol g 2.1 protein is a hydrophobic carrier protein, which can release the compounds through targeted cells with pH gradients, especially cancer cells. Further, we believe that Sol g 2.1 protein can be developed as a drug-delivery protein^52^.

Overall, our comprehensive investigation delves into the intricate pH-dependent binding preferences and conformational dynamics exhibited by the Sol g 2.1 protein when interacting with our selected potential ligands. Unraveling the molecular intricacies governing the interactions between Sol g 2.1 protein and its ligands holds paramount importance in deciphering its physiological roles and exploring its potential applications in targeted drug strategies^25,26,53,54^. This study significantly augments our comprehension of protein–ligand interactions and elucidates the mechanisms underlying their release, thereby furnishing invaluable insights for the development of innovative ligands tailored to target Sol g 2.1 protein for drug delivery purposes^54^^‒^^56^. Furthermore, our findings pave the way for future investigations aimed at elucidating the functional significance of protein-drug carriers in targeting specific cells both in vitro and in vivo, complemented by in silico studies.

## Materials and methods

### Sol g 2.1 protein production

The recombinant protein (rSol g 2.1) was synthesized using a prokaryotic expression system (*E. coli* BL21 (DE3) pLysS, Promega, Selangor, Malaysia) and subsequently purified via affinity chromatography following Nonkhwao et al^3^. Following purification, the rSol g 2.1 protein was separated from other proteins by using the increased concentration of imidazole on the AKTAprime plus system (GE Healthcare). Next, the protein underwent desalination through dialysis and delipidated before being lyophilized and stored at ‒70 °C.

## Competitive binding assay

The FCBA is that a fluorescence probe, N-phenyl-1-naphthylamine (1-NPN, purity > 98%, Sigma Aldrich) binds with the hydrophobic binding cavity of the protein, especially with Tryptophan amino acid (Trp). Then Trp will transfer energy to 1-NPN-probe, leading to an increased emission wavelength at approximately 400 nm with dose-dependent. Besides, when the aliquot of a ligand competitor, the ligand will be competed for 1-NPN-probe, resulting in a decreased emission wavelength at 400 nm. In this study, we performed the FCBA to determine the binding activity of Sol g 2.1 protein and the potential ligands. To examine affinity binding, a stock solution of 5 mM 1-NPN in methanol (HPLC grade) was gradually added to a solution containing 2 µM rSol g 2.1 in 50 mM Tris–HCl buffer (pH 5.5 and pH 7.4) with 1% tween 20. Final concentrations of 0, 0.5, 1, 2, 4, 6, 8, 10, and 12 µM of 1-NPN were achieved, with methanol serving as a negative control^22^. Fluorescence intensity was measured using a spectrofluorometer (FluoroMax + SpectroFluorometer, Horiba Scientific) with excitation at 337 nm and emission scanning from 370 to 490 nm, at room temperature, in triplicate. The equilibrium dissociation (K_d_) value was determined by plotting the fluorescence intensity at 400 nm against each 1-NPN concentration. The data were fitted to a one-site-specific binding model as follows: Y = B_max_*X/(K_d_ + X), where Y, X, B_max_, and K_d_ are the fluorescence intensity (counts/second), fluorescence probe concentration (μM), the maximum specific binding in the same units as Y, and the equilibrium dissociation constant in the same units as X.

The potential ligands, including (*E*)-β-Farnesene, α-Caryophyllene, and 1-Octen-3-ol (all, Tokyo Chemical Industry (TCI), Tokyo, Japan) were used as competitors in the FCBA. For this, a mixture of 2 µM rSol g 2.1 protein and 5 µM 1-NPN in 50 mM Tris–HCl buffer (pH 5.5 and pH 7.4) with 1% tween 20 was titrated with each ligand (5 mM stock in methanol) to final concentrations of 0, 2, 4, 6, 8, 10, 12, 14, 20, 24, 28, and 32 µM. The fluorescence intensity at 400 nm for each concentration was fitted to the One Site-Fit K_i_ model to determine the dissociation constant of competitor ligands (K_i_) as follows: logEC_50_ = log (10^logK_i_*(1 + RadioligandNM/HotK_d_NM))^22,57^. Here, RadioligandNM represents the concentration of fixed 1-NPN (nM), HotKdNM represents K_d_ (nM), and K_i_ represents the equilibrium dissociation constant (Molar). All experiments were conducted in triplicate and analyzed using GraphPad Prism 9 software (San Diego, CA, USA)^22^.

## Molecular docking

The homology model of the Sol g 2.1 protein, derived from the crystallized Sol i 2 structure (*Solenopsis invicta*, PDB ID: 2ygu.1.A, resolution of 2.60 Å), was constructed using the SWISS-MODEL program (https://swissmodel.expasy.org/, accessed on 20 January 2022). Docking simulations of Sol g 2.1 with various ligands were performed using MOE version 2019 (Molecular Operating Environment). Prior to docking, the three-dimensional structural (3D) model of Sol g 2.1 protein underwent protonation using MOE protonate 3D, adjusting its ionization state and adding hydrogen atoms as outlined in previous studies^40,58^.

## Molecular dynamics simulation

Molecular dynamics simulations of complexes between the Sol g 2.1 protein and ligands, such as (*E*)-β-Farnesene, α-Caryophyllene, and 1-Octen-3-ol, were conducted using the GROMACS 5.1.4 package with the Amber99sb Force Field^59^. Initially, the protein–ligand complexes were prepared at pH 7.4 and 5.5 solutions using Protein Preparation for protonation. The protonate 3D step proves to be an effective instrument capable of determining ionization states and accurately placing hydrogen atoms within a macromolecular structure, utilizing their three-dimensional coordinates^60^. Next, an orthorhombic box containing TIP3P water molecules was chosen during the topology process. To achieve a neutralized salt concentration of 0.15 M, sodium (Na^+^) and chloride (Cl^‒^) ions were introduced. The MD simulations utilized an ensemble with the NVT/NPT model, employing a Nose–Hoover chain thermostat to maintain a constant temperature of 300 K and the Martyna-Tobias-Klein-Barostat method to maintain a constant pressure of 1.0 bar^61^. A production run of 100 ns was performed, with 5001 frames saved for analysis.

## Molecular dynamics analysis

The trajectory file generated from the simulation was utilized to compute various structural parameters, including Root Mean Square Deviation (RMSD) and Root Mean Square Fluctuations (RMSF)^62^. The binding free energy (Δ*G*_*MM-PBSA*_) of the ligands with the target protein over the simulation time was computed using g_mmpbsa tool the Molecular Mechanics Poisson-Boltzmann Surface Area (MM-PBSA) within Gromacs^63^. Binding free energy (∆*G*_*bind*_) was calculated using the interaction entropy (IE) method^37^, as outlined below.

For each frame, calculate free binding energy from the interaction energy of the complex and the sum of the interaction energies of the receptor and ligand (Δ*G*_*MM-PBSA*_) as follows:

$${\Delta E}_{pl}^{int}=\text{ Total complex }- (\text{Total receptor }+\text{ Total ligand})$$

Each frame was computed the exponential average of the energy fluctuations as follows:

$${{e}^{\beta \Delta E\genfrac{}{}{0pt}{}{int}{pl}}}=\frac{1}{N}{\sum }_{i=1}^{N}{{e}^{\beta \Delta E\genfrac{}{}{0pt}{}{int}{pl}}\left(ti\right)}$$

Here, ​*β* = 1/*κ*_*B*_*T*, where ​*κ*_*B*_ is the Boltzmann constant and *T* is the temperature. 

Next, compute *–TΔS* to calculate the interaction entropy as follows:

$$-T\Delta S=KBTIn\left({e}^{\beta \Delta E\genfrac{}{}{0pt}{}{int}{pl}}\right)$$

Finally, calculate ∆*G*_*bind*_ from the sum up of Δ*G*_*MM-PBSA*_ and interaction entropy (*–TΔS*).

Additionally, the trajectory analyses began with the Gromacs, and the graphical analyses were performed using the QtGrace (version 0.2.6). The simulation data into the reduced dimensional space of all conformations, we developed a two-component PCA model. This model calculates the principal components over the entire 100 ns simulation, focusing on the alpha carbon chain. The alignment-dependent PCA utilizes Cartesian coordinates^33^. The pKa predictions and protonation fractions for acidic and basic residues in Sol g 2.1 protein complexes with (*E*)-β-Farnesene, α-Caryophyllene, and 1-Octen-3-ol were determined from a 100 ns simulation conducted at pH 7.4 and pH 5.5. Theoretical pKa values, predicted using PDB2PQR software were presented as pKa values (https://server.poissonboltzmann.org/pdb2pqr, accessed on 18 May 2024).

## Supplementary Information

Supplementary Information.

## Data Availability

Supporting information is available in the supplementary file and further supporting data is available from the corresponding author on request.
