# Supplementary material for: Revealing the pH-dependent conformational changes in sol g 2.1 protein and potential ligands binding
Source: Sci Rep. 2024 Sep 11;14:21179. doi: 10.1038/s41598-024-72014-w (PMC11391043; doi:10.1038/s41598-024-72014-w)
Supplement: Supplementary file 1 — Supplementary Information. [file 41598_2024_72014_MOESM1_ESM.doc]

**Revealing the pH-Dependent Conformational Changes in Sol g 2.1 Protein and Potential Ligands Binding**

Siriporn Nonkhwao ^1,4^; Doungkamol Leaokittikul ^1^, Rina Patramanon ^2,4^, Nisachon Jangpromma ^2,4^, Jureerut Daduang ^3^, Sakda Daduang ^1,4*^

^1^ Faculty of Pharmaceutical Sciences, Khon Kaen University, Khon Kaen, 40002, Thailand

^2^ Department of Biochemistry, Faculty of Science, Khon Kaen University, Khon Kaen, 40002, Thailand

^3^ Faculty of Associated Medical Sciences, Khon Kaen University, Khon Kaen 40002, Thailand

^4^ Protein and Proteomics Research Center for Commercial and Industrial Purposes (ProCCI), Khon Kaen University, Khon Kaen, 40002, Thailand

* Corresponding author: Tel.: +6643-202-378, E-mail: [sakdad@kku.ac.th](mailto:sakdad@kku.ac.th)


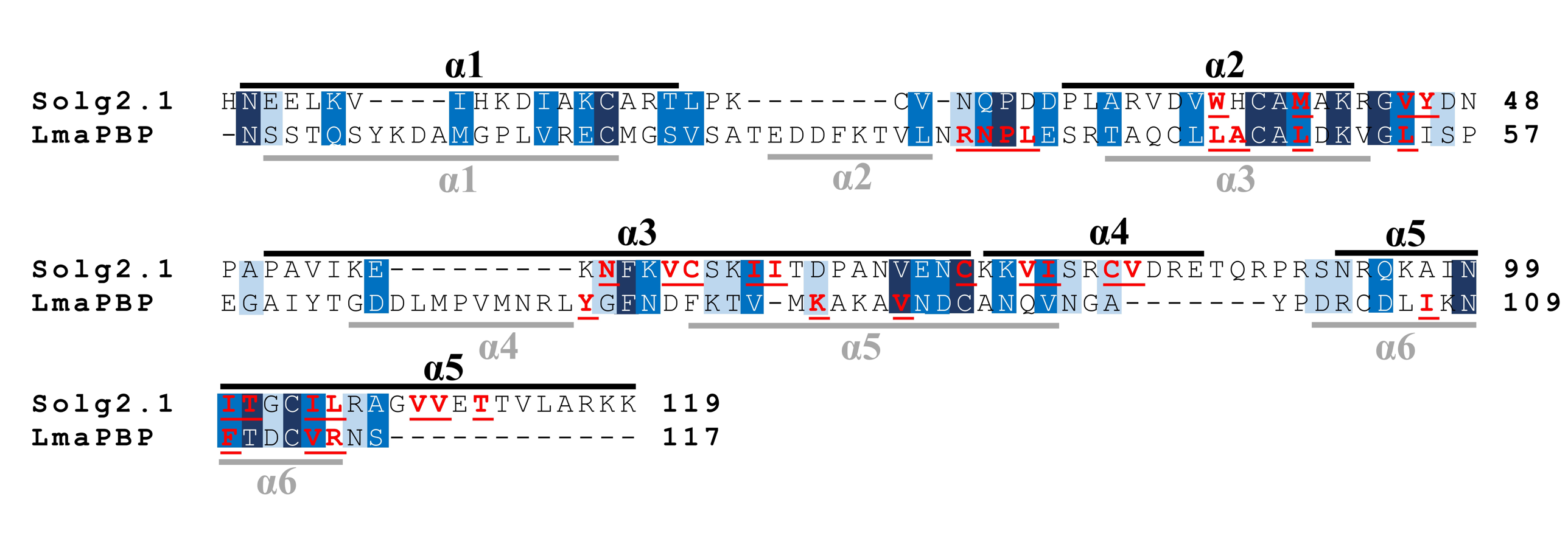


**Figure S1.** The sequence alignment between Sol g 2.1 (GenBank: UYX46120.1) and LmaPBP (below; PDB ID: 1org.1.A). The α-helices were denoted by the upper black line (Sol g 2.1) and lower gray line (LmaPBP). Identical residues are highlighted with a blue background. Residues with similar physicochemical properties and groups were depicted within a medium and pale blue, respectively. Letter with underline indicated inner cavity binding residue.


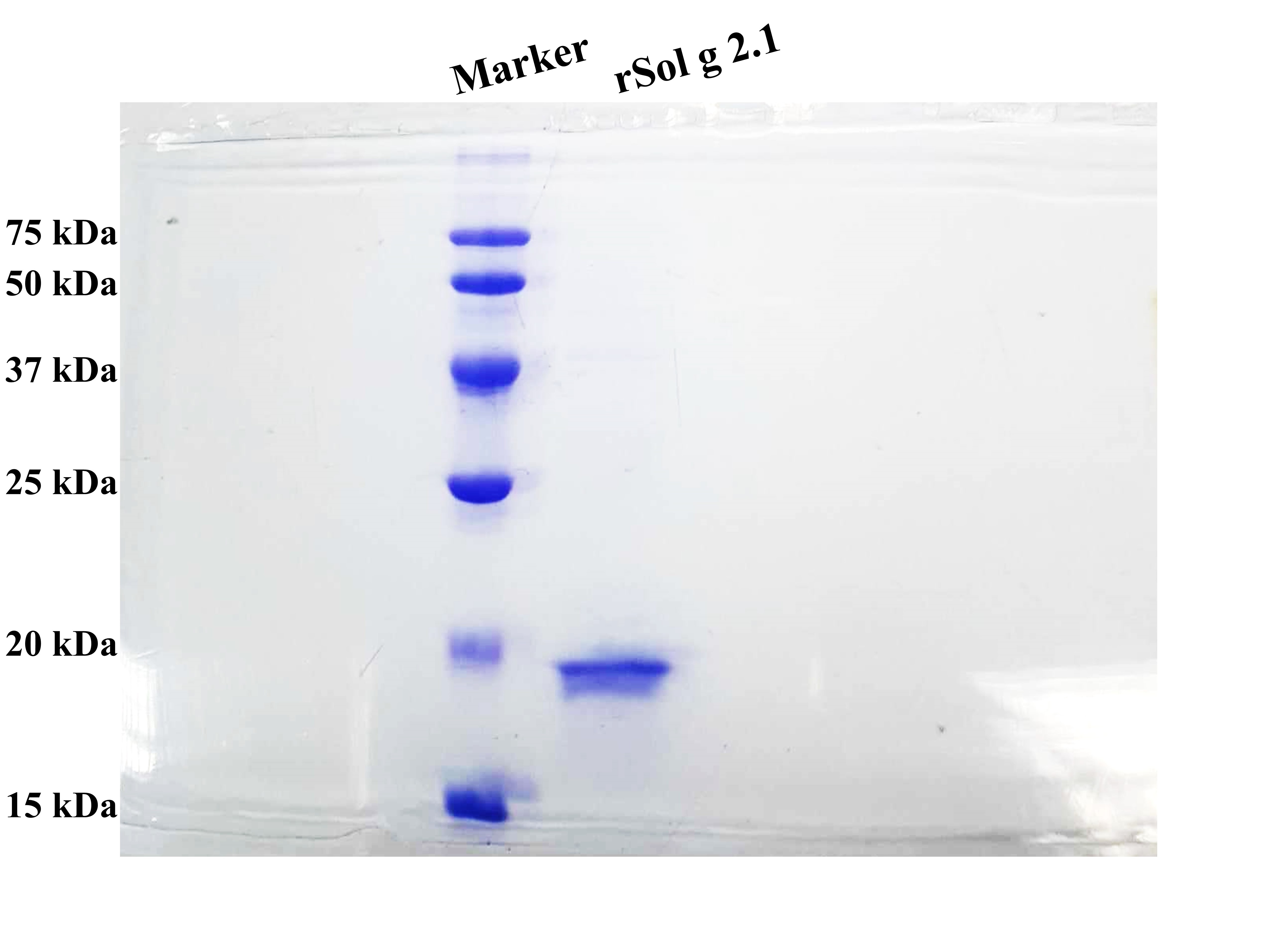


**Figure S2.** SDS-PAGE image shows the purified rSol g 2.1 protein (molecular weight ~17 kDa), produced in *E. coli* BL21, and purified using ion-exchange gradient elution on an AKTAprime plus system.





**Figure S3.** The depletion of fluorescence emission spectra that were recorded in the 370-490 nm wavelength range. **(A)**, **(C)**, and **(E)** Show the depletion of fluorescence spectra of rSol g 2.1 protein and (*E*)-β-Farnesene, α-Caryophyllene, and 1-Octen-3-ol at pH 7.4, respectively. **(B)**, **(D)**, and **(F)** Show the depletion of fluorescence spectra of rSol g 2.1 protein and (*E*)-β-Farnesene, α-Caryophyllene, and 1-Octen-3-ol at pH 5.5, respectively.





**Figure S4.** Sol g 2.1 protein and ligand interactions mapping from MOE docking of (*E*)-β-Farnesene, α-Caryophyllene, and 1-Octen-3-ol at pH 7.4 and 5.5.
